# Effects of *Spartina alterniflora* invasion on the communities of methanogens and sulfate-reducing bacteria in estuarine marsh sediments

**DOI:** 10.3389/fmicb.2013.00243

**Published:** 2013-08-23

**Authors:** Jemaneh Zeleke, Qiang Sheng, Jian-Gong Wang, Ming-Yao Huang, Fei Xia, Ji-Hua Wu, Zhe-Xue Quan

**Affiliations:** ^1^Department of Microbiology and Microbial Engineering, School of Life Sciences, Fudan UniversityShanghai, China; ^2^Ministry of Education Key Laboratory for Biodiversity Science and Ecological Engineering, Department of Ecology and Evolutionary Biology, Fudan UniversityShanghai, China

**Keywords:** dissimilatory sulfite reductase B (*dsrB*), methyl coenzyme M reductase A (*mcrA*), *spartina alterniflora*, *phragmites australis*, estuarine marsh

## Abstract

The effect of plant invasion on the microorganisms of soil sediments is very important for estuary ecology. The community structures of methanogens and sulfate-reducing bacteria (SRB) as a function of *Spartina alterniflora* invasion in *Phragmites australis-vegetated* sediments of the Dongtan wetland in the Yangtze River estuary, China, were investigated using 454 pyrosequencing and quantitative real-time PCR (qPCR) of the methyl coenzyme M reductase A (*mcrA*) and dissimilatory sulfite-reductase (*dsrB*) genes. Sediment samples were collected from two replicate locations, and each location included three sampling stands each covered by monocultures of *P. australis, S. alterniflora* and both plants (transition stands), respectively. qPCR analysis revealed higher copy numbers of *mcrA* genes in sediments from *S. alterniflora* stands than *P. australis* stands (5- and 7.5-fold more in the spring and summer, respectively), which is consistent with the higher methane flux rates measured in the *S. alterniflora* stands (up to 8.01 ± 5.61 mg m^−2^ h^−1^). Similar trends were observed for SRB, and they were up to two orders of magnitude higher than the methanogens. Diversity indices indicated a lower diversity of methanogens in the *S. alterniflora* stands than the *P. australis* stands. In contrast, insignificant variations were observed in the diversity of SRB with the invasion. Although *Methanomicrobiales* and *Methanococcales*, the hydrogenotrophic methanogens, dominated in the salt marsh, *Methanomicrobiales* displayed a slight increase with the invasion and growth of *S. alterniflora*, whereas the later responded differently. *Methanosarcina*, the metabolically diverse methanogens, did not vary with the invasion of, but *Methanosaeta*, the exclusive acetate utilizers, appeared to increase with *S. alterniflora* invasion. In SRB, sequences closely related to the families *Desulfobacteraceae* and *Desulfobulbaceae* dominated in the salt marsh, although they displayed minimal changes with the *S. alterniflora* invasion. Approximately 11.3 ± 5.1% of the *dsrB* gene sequences formed a novel cluster that was reduced upon the invasion. The results showed that in the sediments of tidal salt marsh where *S. alterniflora* displaced *P. australis*, the abundances of methanogens and SRB increased, but the community composition of methanogens appeared to be influenced more than did the SRB.

## Introduction

Estuarine wetlands are among the most productive ecosystems on the Earth and provide many key ecosystem services. These environments are highly vulnerable to the invasion of exotic plant species, and ecosystem functions may be altered as a consequence of the invasion (Williams and Grosholz, 2008). For example, the native *Phragmites australis* and *Scirpus mariqueter* communities of the Dongtan tidal flats, comprising approximately 32,600 ha of the Yangtze River estuary, are currently being invaded by the aggressive exotic *Spartina alterniflora.* Furthermore, *S. alterniflora* was introduced to the Yangtze River estuary in 1990s to increase the protection of coastal banks and to accelerate sedimentation and land formation (Chung, 1993; Liao et al., 2007). However, due to its extensive expansion (approximately 43% of the vegetated part as of 2004) (Chen et al., 2008) and displacement of the native species, a number of ecological impacts have occurred. The impacts of *S. alterniflora* invasion on the aboveground flora and fauna of Dongtan tidal salt marsh have been described by several studies (Jiang et al., 2009; Zhang et al., 2010). Recently, a study by Cheng and his colleagues (2007) demonstrated that methane flux rates in *S. alterniflora* stands are higher than in areas dominated by *P. australis*, which is consistent with several studies that indicate higher methane flux rates in the stands covered by *S. alterniflora* than in those covered by other salt marsh plants (Cheng et al., 2007; Wang et al., 2009; Zhang et al., 2010; Tong et al., 2012). The root exudates and litter decomposition of *S. alterniflora* can alter the nutritional contents of soils, which in turn may affect the functioning of soil microbial communities. Hence, parallel to the impacts that *S. alterniflora* may pose to the aboveground ecology, it has the potential to affect belowground microbial communities and processes. In tidal marshes of estuaries where large amounts of vegetation and river-derived organic matter are deposited, nutrients are typically mineralized through anaerobic processes, predominantly via methanogenesis and sulfate reduction.

Methanogenesis and dissimilatory sulfate reduction are the two key terminal electron-scavenging processes in the anaerobic decomposition of organic matter. These anaerobic processes are known to compete for hydrogen and acetate (Abram and Nedwell, 1978; King, 1984), but the latter has higher affinity and can outcompete with methanogenesis in sediments having high sulfate concentration, such as marine and salt marsh surface sediments (Oremland et al., 1982; Winfrey and Ward, 1983; Purdy et al., 2003). Although competition is one of the major factors controlling the distribution and activity of methanogenesis, land and tide-derived depositions and autochthonous production by marsh plants and freshwater dilutions of sea water in tidal marshes may create conditions for methanogenesis to be a major terminal electron accepting process (Senior et al., 1982; Schubauer and Hopkinson, 1984; Holmer and Kristensen, 1994; Kaku et al., 2005). As the type of marsh plants may determine the availability and level of nutrients in sediments and, hence, microbial activities (Andersen and Hargrave, 1984; Ravit et al., 2003; Hawkes et al., 2005; Batten et al., 2006), the structure and function of methanogens and SRB in salt marshes may be influenced by the invasion and displacement of native plants by exotic species.

Methanogens are strictly anaerobic microorganisms. They are composed of six well-established archaeal orders: *Methanobacteriales, Methanococcales, Methanomicrobiales, Methanosarcinales, Methanocellales*, and *Methanopyrales*. Moreover, recent culture independent techniques have discovered novel methanogen members such as Zoige cluster-I (ZC-I) (Zhang et al., 2008) and the anaerobic methanotrophs (ANME-1, 2 and 3) (Knittel et al., 2005). Most methanogens are hydrogenotrophic, utilizing carbon dioxide as a source of carbon and hydrogen or formate as electron sources. However, members of the genus *Methanosarcina* are physiologically versatile, additionally utilizing acetate and methylated compounds such as methanol, monomethylamine, dimethylamine and trimethylamine, whereas genus *Methanosaeta* is restricted to acetate (Liu and Whitman, 2008). Similarly, sulfate-reducing microbes are strictly anaerobic microorganisms. They reduce sulfate to sulfide using several substrates such as hydrogen, formate, acetate, butyrate, propionate and ethanol as electron sources (Barton and Fauque, 2009). These microbes are found within several phylogenetic lines: in the class *Deltaproteobacteria* (*Desulfobacterales, Desulfovibrionales*, and *Syntrophobacterales*), phylum *Firmicutes* (*Desulfotomaculum, Desulfosporomusa*, and *Desulfosporosinus*), phylum *Nitrospirae* (*Thermodesulfovibrio*), phylum *Thermodesulfobacteria* and archaeal genera (*Archaeoglobus, Thermocladium*, and *Caldivirga*). Diverse phylogenetic lines generally present major challenges to specifically targeting these organisms in environmental samples using the universal 16S rRNA gene as a marker. Therefore, targeting group-specific functional genes, such as those encoding methyl coenzyme M reductase A (*mcrA*) and dissimilatory sulfite reductase (*dsrB*), are commonly used as alternatives to the 16S rRNA gene to investigate methanogens and SRB, respectively (Luton et al., 2002; Dar et al., 2006; Geets et al., 2006).

In this study, 454 pyrosequencing and quantitative real-time PCR (qPCR) were used to investigate the diversity and abundance of methanogens and SRB in the sediments vegetated by *P. australis, S. alterniflora* and transition stands where both plants are available. Investigations were conducted in two seasons: before growth (April) and during the full growth stage (August).

## Materials and methods

### Sampling and *in situ* measurements

This study was conducted in the tidal salt marsh of Dongtan, where the intentionally introduced *S. alterniflora* is aggressively displacing the native *P. australis*. Two replicate sampling locations (approximately 60 m apart) were selected, each with three distinct sampling stands (approximately 20 m apart) covered by monocultures of *P. australis* (non-invaded), transition stands (both plants available) and *S. alterniflora* (completely displaced). Investigations were conducted in two seasons: before growth (April) and at full growth stage (August). In both sampling seasons, the methane and carbon dioxide flux rates were determined from each location by collecting the gases using the enclosed static chamber technique (Hirota et al., 2004) and gas chromatographic analysis using a 6890N gas chromatograph (Agilent Technologies, Ltd. USA).

Soil temperatures and conductivities were directly measured using a Field Scout™ direct soil EC meter with a jab probe (2265FS, USA), whereas pH was measured using a IQ150 portable pH meter (IQ Scientific Instruments, USA). From each sampling stand, five replicate sediment samples (surface to 5 cm) were collected within a radius of approximately 2 m. Soil samples were immediately sealed in polyethylene bags and transported to the laboratory on ice. Within one hour of collection, replicate samples were homogenized and stored at −20°C for downstream analyses.

Sediment samples for chemical analysis were dried completely in an oven at 50°C. Then, all non-decomposed plant litter and root materials were removed easily from the gently crushed sediments. The sediments were then ground into powder and passed through #10 meshes where part of the powder was used for determination of total carbon and total nitrogen using an NC analyzer (FlashEA1112 Series, Thermo Inc., Italy), and the remainder was used for analysis of sulfate ions using an ion chromatograph (ICS-1000; Dionex, USA).

### DNA extraction and PCR amplification

The total genomic DNA of each sample was extracted in duplicate tubes from 0.25 g of sediment (wet weight) using a PowerSoil DNA Kit (Mo Bio Laboratories, USA) following the manufacturer's instructions. Amplicons for 454 pyrosequencing were prepared following the 2-step barcoded PCR method (Berry et al., 2011). In the first-step PCR, the 50 μl reaction contained 2 μl of template DNA (5–10 ng), 25 μl of Taq PCR Master Mix (TianGen, China), 2 μl (10 μM) of each primer (Table 1), 2 μl of bovine serum albumin (BSA) (0.8 μg ul^−1^ final concentration) and 17 μl of distilled water. Amplifications were conducted in a thermal cycler (PCR Thermal Cycler Dice; Takara, Japan) for 25 cycles. For the second-step PCR, the forward and reverse primers were modified as follows: 5′-Adapter A + 8 bp barcode + TC + forward primer—3′ and 5′-Adapter B + CA + reverse primer −3′, respectively. Whereas Adaptor A and B represent the 30 bp that were used as sequencing primers, the 8-bp barcode sequences were used to identify individual samples, and TC and CA were used as linkers. For each sample, 2 μl of the first-step PCR product was used as template DNA in the second-step PCR. Except for the annealing temperatures, which were raised by 2°C, all the PCR conditions were the same in the second-step PCR as in the first-step PCR. Amplifications were conducted for 10 cycles. The PCR products were then purified using the UltraClean PCR Clean-Up kit (MoBio, USA) and then quantified using the PicoGreen reagent (Invitrogen, USA) for dsDNA on a ND3300 Fluorospectrometer (NanoDrop Technologies, USA). Lastly, the amplicons of all samples were pooled in an equivalent concentration for 454 pyrosequencing. Pyrosequencing was conducted using a Roche/454 (GS FLX Titanium System). For 454 pyrosequencing, the barcoded and pooled amplicons were rechecked for the absence of primer dimers, and emulsion PCR was set up according to Roche's protocols.

**Table 1:** **Primers used for pyrosequencing and/or qPCR**.

**Primer**	**Sequence (5′ – 3′)**^**a**^	**Target**	**Ta (°C)**	**References**
Mlas	GGTGGTGTMGGDTTCACMCARTA	*mcrA* gene	55	Steinberg and Regan, 2008
mcrA-rev	CGTTCATBGCGTAGTTVGGRTAGT			
DSRp2060F	CAACATCGTYCAYACCCAGGG	*dsrB* gene	53	Dar et al., 2006; Geets et al., 2006
Dsr-4Rdeg	GTGTARCAGTTDCCRCA			
27f	AGAGTTGATYMTGGCTCAG	Bacterial 16S rDNA (for pyrosequencing)	52	Giovannoni et al., 1988
536r	GTATTACCGCGGCKGCTG			
338f	ACTCCTACGGGAGGCAGC	Bacterial 16S rDNA (for qPCR)	52	Giovannoni, 1991
536r	GTATTACCGCGGCKGCTG			
Arch340F	CCCTAYGGGGYGCASCAG	Archaeal 16S rDNA	57	Gantner et al., 2011
Arch1000R	GAGARGWRGTGCATGGCC			

a M, A/C; D, A/G/T; R, A/G; B, C/G/T; V, A/C/G; Y, C/T; K, G/T; S, G/C; W, A/T.

### Quantitative real-time PCR

SYBR Green I-based qPCR was conducted for both the 16S rRNA gene (bacteria and archaea) and functional genes (*mcrA* and *dsrB*) using the primers presented in Table 1. The coverage and specificity of the functional gene primers were validated through clone library construction before application to qPCR. The reaction mixes were prepared as previously described (Zeleke et al., 2013), and triplicate reaction tubes were used for each sample. Known copy numbers of linearized plasmid DNA with the respective gene inserted from pure clones were used as standards for the quantifications. The linearization of the plasmid DNA of each gene was performed using the *Eco*RI restriction enzyme (Fermentas, USA) following the recommended protocol. The amplification efficiency and R^2^ values were between 90–99% and 0.98–1, respectively. qPCR of all genes were conducted using a MX3000P QPCR thermocycler (Stratagene, USA). The annealing temperatures are described in Table 1. Melting curves were analyzed to detect the presence of primer dimers. The results were analyzed using MxPro QPCR software version 3.0 (Stratagene, USA).

### Data analysis

The raw 454 pyrosequencing data of the 16S rRNA and functional genes were trimmed using the sample-specific barcodes, in which the forward primers and sequences with ambiguous nucleotides were removed. For the functional genes, BLASTx analyses were performed against the known sequences of the NCBI database. The sequences that did not match the target gene were excluded from further analysis. The remaining sequences were translated by the RDP's functional gene and repository FrameBot tool (http://fungene.cme.msu.edu/FunGenePipeline/framebot/form.spr), which detects and corrects the likely frameshift errors. After removing amino acid sequences with stop codons and/or unknown amino acids, the remaining sequences were aligned by the RDP's functional gene and repository aligner tool. A few (0.5–1%) poorly aligned amino acid sequences were also removed, and the names of the remaining quality amino acid sequences were used to recover the corresponding original nucleotide sequences using Mothur software, version 1.8 (Schloss et al., 2009). Moreover, potential chimeric sequences were also removed (<1%) using the chimra.uchime command in Mothur (Schloss et al., 2009). The final quality nucleotide sequences were used to define operational taxonomic units (OTUs) and downstream analyses. Before calculating the diversity indices, the sequence numbers of each sample were normalized to an equal number. Sequence information was also used for principal component analysis (PCA) using the UniFrac online tool (http://bmf.colorado.edu/unifrac/). For 16S rRNA genes of the total bacteria and archaea, raw data sequences were treated through the Mothur program (Schloss et al., 2009). Briefly, after trimming, pre-clustering and removing the potential chimeric sequences, the remaining purified sequences were used for phylogenetic affiliation, which was performed through BLAST analysis against the Silva taxonomy files at an 80% threshold value.

### Sequence accession numbers

All the *mcrA, dsrB* and 16S rRNA genes of the total bacterial and archaeal sequences recovered from the estuarine marsh of Dongtan were deposited in the NCBI's sequence read archives with the accession numbers of SRP021055, SRP021326, SRP021327 and SRP021329, respectively.

## Results

### Methane flux rates and sediment characteristics

**Figure 1:**
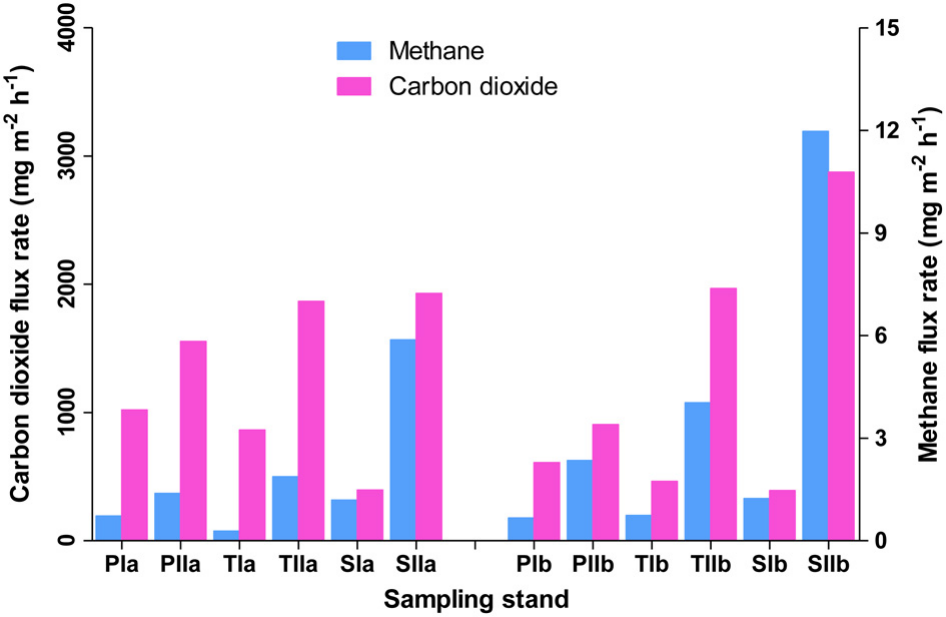
**Methane and carbon dioxide flux rates in the *P. australis* (P), *S. alterniflora* (S) and transition (T) stands in the Dongtan salt marsh located in the Yangtze River estuary**. In the sample names, I and II represent the spring and summer samples, respectively, whereas “a” and “b” indicate the replicate locations.

Methane flux rates differed both with the invasion and growth of *S. alterniflora* (Figure 1). In spring, the mean flux rates in the *P. australis*, transition, and *S. alterniflora* stands were approximately 0.51 ± 0.31, 0.93 ± 0.37, and 0.99 ± 0.35 mg m^−2^ h^−1^, respectively. This indicates an approximately 97% increase of flux rate with *S. alterniflora* invasion. When the plants were fully grown, these flux rates were increased to 1.63 ± 0.34, 4.11 ± 2.49, and 8.01 ± 5.61 mg m^−2^ h^−1^, respectively, in the *P. australis*, transition and *S. alterniflora* stands and the impact of *S. alterniflora* was significant in the summer (152 and 391% higher in the transition and *S. alterniflora* strands, respectively, than in *P. australis* stands). The gas flux rates also displayed significant increases in the summer (225, 343, and 709% in the *P. australis*, transition and *S. alterniflora* stands, respectively). These increases were positively correlated with the change in temperatures (*R*^2^ = 0.2, α = 0.05), although not significant. Similarly, carbon dioxide flux rates were relatively high in the *S. alterniflora* stands. When the mean flux ratios of methane to carbon dioxide were compared, higher values were observed in the *S. alterniflora* stands than in the *P. australis* stands. In spring, the ratios were approximately 9 × 10^−4^ and 3 × 10^−3^, in the *P. australis* and *S. alterniflora* stands, respectively, representing an approximate 3.5-fold increase in the *S. alterniflora* stands (Figure 1). In summer, these ratios were approximately 1.74 × 10^−3^ and 3.6 × 10^−3^, in the *P. australis* and *S. alterniflora* stands, respectively, representing an increase of approximately 2.1-fold in the *S. alterniflora* stands.

As expected, soil temperature, pH, conductivity and sulfate levels did not vary across the sampling stands (Table 2). However, there was a marked increase in the mean soil temperature from spring to summer (from approximately 15—28°C). In both seasons, the total carbon (TC) and total nitrogen (TN) levels of sediments were higher in the *S. alterniflora*-influenced sediments than the *P. australis* sediments. However, minor reductions of TC and TN were observed in all stands during the summer (Table 2).

**Table 2:** **Characteristics of sediments from *P. australis* (P), *S. alterniflora* (S) and transition (T) stands in the Dongtan salt marsh in the Yangtze estuary**.

**Sample**	**pH**		**Temperature (°C)**		**Conductivity (mS)**		**Sulfate (mg kg ^**−1**^)**		**TC (% of dry soil)**		**TN (% of dry soil)**	
	**Spring**	**Summer**	**Spring**	**Summer**	**Spring**	**Summer**	**Spring**	**Summer**	**Spring**	**Summer**	**Spring**	**Summer**
Pa	6.1 ± 0.1	7.1 ± 0.1	15.4 ± 0.9	27.9 ± 0.1	ND	7.8 ± 0.6	242.4	272.2	2.1	2	0.14	0.14
Pb	5.9 ± 0.1	7.1 ± 0.1	14.8 ± 0.2	28.1 ± 0.1	7.5 ± 1.2	7.3 ± 0.2	224.7	275.4	2.4	2	0.21	0.14
Ta	6.2 ± 0.2	7.1 ± 0.1	15.9 ± 0.4	27.5 ± 0.2	ND	8.2 ± 1.1	287.3	331	2.7	2.5	0.19	0.18
Tb	5.9 ± 0.1	7.3 ± 0.4	16.3 ± 0.3	28.1 ± 0.1	6.6 ± 0.5	7.7 ± 0.5	245.3	254.7	3.1	2.6	0.22	0.18
Sa	5.8 ± 0.2	6.9 ± 0.1	15.5 ± 0.4	27.8 ± 0.2	ND	9.4 ± 0.7	263.4	331.8	3.3	3.5	0.25	0.23
Sb	6.2 ± 0.2	7.4 ± 0.4	13.5 ± 0.4	27.9 ± 0.1	8.2 ± 0.8	7.4 ± 0.3	326.6	325	3.5	3.3	0.24	0.22

In the sample names, ‘a’ and ‘b’ represent the replicate sampling locations.

Values given for pH, temperature and conductivity are the means ± standard deviations of 5 replicate measurements, but sulfate was measured once (from the mixed samples) in the lab. ND, not determined.

### Abundances of methanogens and SRB

**Figure 2:**
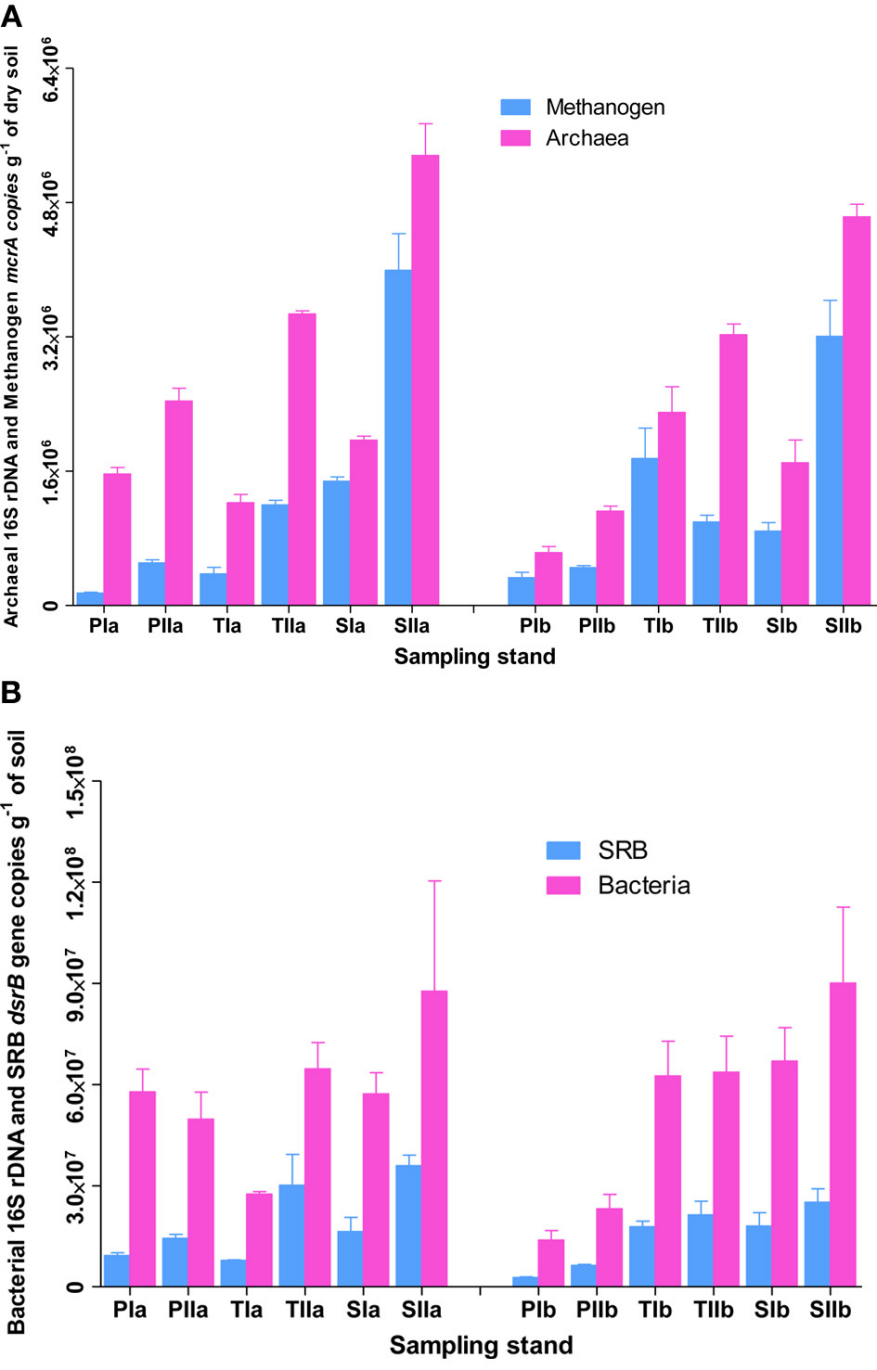
**Abundance of (A) methanogens and (B) SRB in sediments from *P. australis* (P), *S. alterniflora* (S) and transition (T) stands of the Dongtan salt marsh located in the Yangtze River estuary**. The error bars represent the standard deviation of three replicate reaction tubes. The sample names are as described in Figure 1.

To understand the overall relative abundances of methanogens and SRB, SYBR Green I-based quantification of total archaeal and bacterial 16S rRNA genes were determined from the samples used to investigate methanogens and SRB. As revealed from the copy numbers of *mcrA* and *dsrB* genes, the abundances of methanogens and SRB varied with the invasion and growth of *S. alterniflora* (Figures 2A,B). In spring, the mean abundance of methanogens were approximately 2.4 ± 1.3 × 10^5^, 1.1 ± 0.9 × 10^6^ and 1.2 ± 0.4 × 10^6^ copies per g of dried soil in the *P. australis*, transition and *S. alterniflora* stands, respectively, indicating there were approximately 5 times more methanogens in the *S. alterniflora* stands than in the *P. australis* stands. Furthermore, higher abundances of methanogens were observed in the summer (4.8 ± 0.1 × 10^5^, 1.2 ± 0.1 × 10^6^ and 3.6 ± 0.6 × 10^6^ copies per g of dried soil in the *P. australis*, transition and *S. alterniflora* stands, respectively), representing a dramatic increase of methanogens in the *S. alterniflora* stands (approximately 7.5-fold higher than the *P. australis* stands). In terms of the changes associated with *S. alterniflora* invasion, similar trends were observed for the abundance of archaeal 16S rRNA gene copies in both sampling seasons (Figure 2A). The mean abundance proportions of methanogens (copies of *mcrA* to 16S rRNA gene of total archaea) were also higher in the *S. alterniflora*-impacted stands than in the *P. australis* stands. In the spring, the mean abundance proportions of methanogens were approximately 31, 53, and 63% in the *P. australis*, transition and *S. alterniflora* stands, respectively, whereas they slightly changed to 30, 32 and 71%, respectively, in the summer.

Similarly, the invasion and growth of *S. alterniflora* increased the abundance of SRB in both sampling seasons (Figure 2B). In the spring, the mean abundances of SRB were approximately 5.99 ± 4.06 × 10^6^, 1.28 ± 0.71 × 10^7^, and 1.72 ± 0.12 × 10^7^ per g of dried soil, respectively, for *P. australis*, transition and *S. alterniflora* stands, indicating 2.2- and 2.9-times more copies in the transition and *S. alterniflora* stands than in the *P. australis* stands, respectively. Although the abundance of SRB was greater than that of the methanogens (up to 2 orders of magnitude), the increases in the mean abundances of SRB associated with *S. alterniflora* invasion in the summer were relatively lower (approximately 3.0-fold lower than the abundances in the spring). When compared with the total bacterial abundance, the mean abundance proportions of the SRB ranged from 17 to 34%. Unlike most of the sediment characteristics measured at the salt marsh, TC was positively correlated with the abundance of methanogens (*R*^2^ = 0.54, α = 0.05) and SRB (*R*^2^ = 0.70, α = 0.05).

### Methanogens and SRB diversity

Purified *mcrA* sequences were used to define OTUs at the genus (89% similarity cutoff) and family (79% similarity cutoff) levels (Steinberg and Regan, 2008). At the genus level, maximum numbers of *mcrA* OTUs were observed in sediments collected from *P. australis* stands, whereas the minimum numbers were observed in sediments from *S. alterniflora* stands (Table 3). Rarefaction curves at both similarity cutoffs leveled off horizontally between 30 and 58 OTUs (data not shown here), indicating the use of acceptable numbers of sequences for good representation of the methanogen communities from the study site. Despite the greater abundance of methanogens in the *S. alterniflora* stands of both sampling seasons (Figure 2A), richness and diversity indices (Chao1, ACE and Shannon) indicated lower diversity and richness of methanogens in the *S. alterniflora* stands compared with the *P. australis* stands (Table 3).

**Table 3:** **Summary of the diversity indices of *mcrA* genes amplified from sediment samples of the *S. alterniflora* (S), transition (T) and *P. australis* (P) stands**.

**Season**	**Sample**	**NS^**a**^**	**89% Similarity cutoff**				**79% Similarity cutoff**			
			**NO^**b**^**	**Chao1**	**ACE**	**Shannon**	**NO^**b**^**	**Chao1**	**ACE**	**Shannon**
Spring	PIa	162	58	128	188	3.6	33	46	65	2.8
	PIb	176	39	65	115	2.6	22	23	24	2
	TIa	504	39	58	82	2.5	22	31	39	1.9
	TIb	479	32	56	60	2.3	20	33	41	1.7
	SIa	1455	33	60	61	2.4	21	30	32	1.8
	SIb	1035	25	51	148	2	20	31	33	1.6
Summer	PIIa	493	50	95	182	3.3	36	113	163	2.7
	PIIb	319	50	347	371	3.2	26	61	111	2.3
	TIIa	598	34	111	130	2.5	26	44	72	2.1
	TIIb	516	35	50	96	2.7	22	31	41	2.1
	SIIa	763	35	98	97	2.7	20	56	41	2.1
	SIIb	586	41	96	93	2.9	26	37	68	2.3

The diversity indices were calculated at 89 and 79% similarity cutoffs using normalized sequence numbers. The sample names are as described in Figure 1.

NS^a^, number of sequences; NO^b^, number of OTUs.

However, *dsrB* OTUs were defined at three sequence similarity cutoffs (90, 80, and 70%). Unlike *mcrA*, significantly large numbers of *dsrB* OTUs were observed at 90 and 80% similarity cutoffs (Table 4). At the 70% similarity cutoff, the numbers of OTUs were reduced by more than half (approximately 160 OTUs), which was used in the construction of a phylogenetic tree. Despite the diversity of SRB, the rarefaction curves indicated good representation of the SRB, particularly at the 80% similarity cutoff (data not shown here). In both seasons, the diversity indices (Chao1, ACE and Shannon) did not vary significantly with *S. alterniflora* invasion (Table 4), suggesting its minimal impact on the diversity of SRB.

**Table 4:** **Summary of the diversity indices of *dsrB* genes amplified from sediment samples in the *S. alterniflora* (S), transition (T), and *P. australis* (P) stands**.

**Season**	**Sample**	**NS^**a**^**	**90% Similarity cutoff**				**80% Similarity cutoff**			
			**NO^**b**^**	**Chao1**	**ACE**	**Shannon**	**NO^**b**^**	**Chao1**	**ACE**	**Shannon**
Spring	PIa	414	187	421	600	4.7	144	306	356	4.3
	PIb	704	310	1096	2137	5.4	202	456	846	4.8
	TIa	778	294	1277	2605	5.3	203	530	847	4.8
	TIb	706	307	1000	2179	5.3	215	493	874	4.8
	SIa	971	165	331	460	4.7	138	199	215	4.5
	SIb	701	303	1558	3092	5.3	214	584	1073	4.9
Summer	PIIa	523	274	591	989	5.3	193	302	463	4.9
	PIIb	675	285	979	2650	5.3	191	466	1093	4.7
	TIIa	740	311	883	1349	5.5	209	483	680	4.9
	TIIb	686	279	882	1923	5.3	184	455	705	4.7
	SIIa	704	285	1015	2283	5.2	185	407	570	4.6
	SIIb	890	140	324	368	4.6	116	163	164	4.3

The diversity indices were calculated at 90 and 80% similarity cutoffs using normalized sequence numbers. Sample names are as described in Figure 1.

NS^a^, number of sequences; NO^b^, Number of OTUs.

### Phylogenetic analyses of *mcrA* and *dsrB* OTUs

To determine the identity and phylogenetic positions of the collected sequences, trees were constructed for both the methanogens and SRB. OTU representatives of both genes (79 and 70% similarity OTUs for *mcrA* and *dsrB*, respectively) were translated into amino acid sequences, and their closest relatives were searched for using the NCBI amino acid non-redundant database (BLASTp). For SRB, a 70% similarity cutoff was selected because the numbers of OTUs observed at 90 and 80% similarity cutoffs were relatively large (up to 300) with small variation (<6.5%) among their relative composition.

**Figure 3:**
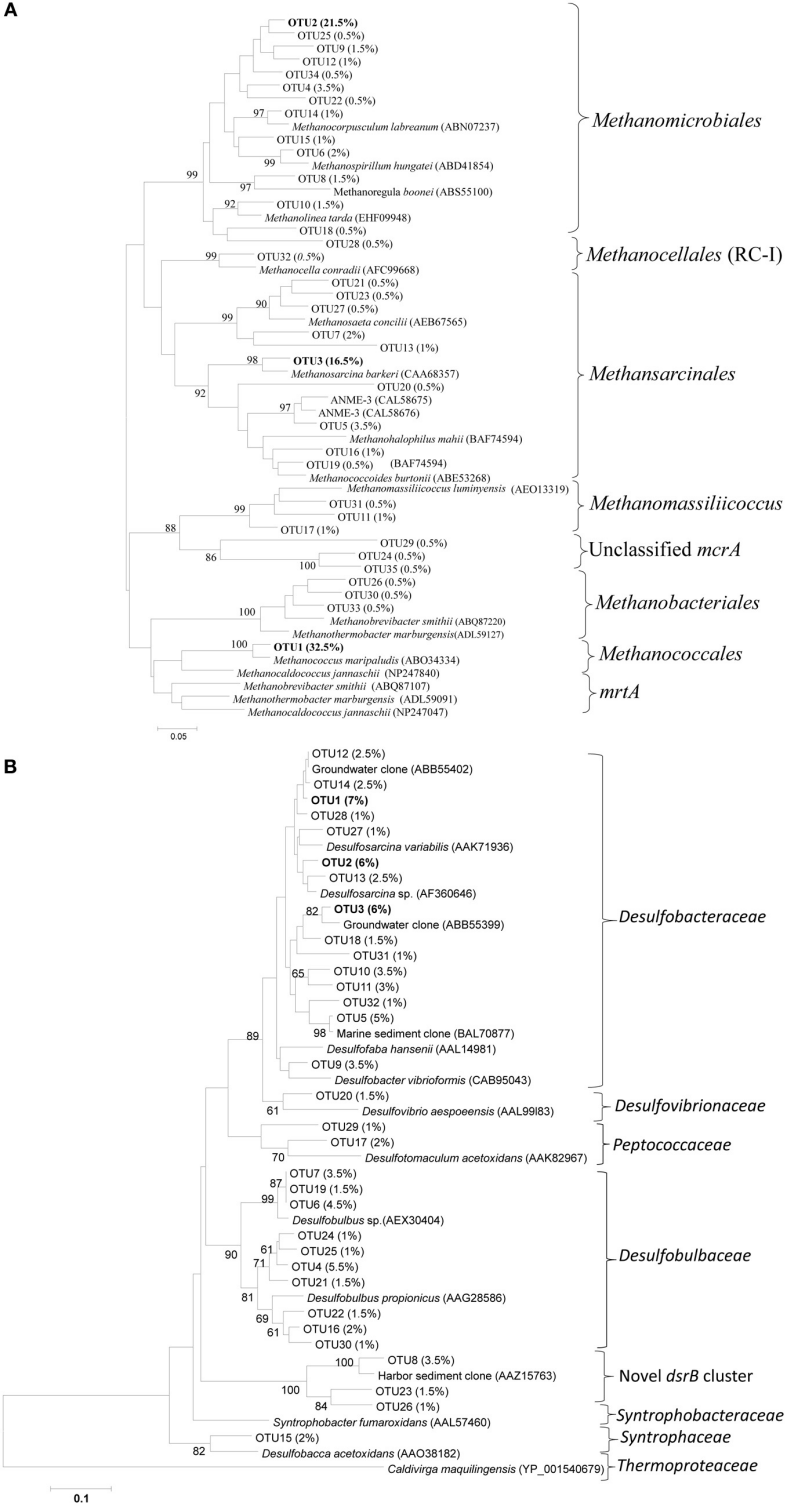
**Neighbor-joining phylogenetic trees of (A) *mcrA* and (B) *dsrB* gene OTUs recovered from the Dongtan tidal salt marsh located in the Yangtze estuary**. The trees were constructed based on the inferred amino acids of the OTU-representative nucleotide sequences. Percentages in the parenthesis indicate the percent of sequences included in that specific OTU. Only OTUs containing at least 0.5 and 1% of the total sequences *mcrA* and *dsrB* OTUs, respectively, are presented here.

*McrA* OTUs were dominated by OTU1, 2 and 3, representing approximately 32.5, 21.5, and 16.5% of the total sequences (Figure 3A). OTU1 was closely related to the *mcrA* of *Methanococcus*, whereas OTU2 and 3 were related to the *mcrA* of *Methanomicrobiales* and *Methanosarcina*, respectively. Most of the *mcrA* gene sequences were closely related to the methanogen orders *Methanomicrobiales, Methanosarcinales* and *Methanococcales, although* other methanogens such as *Methanobacteriales*, ANME (1 and 3) and ZC-I were also detected in the salt marsh (Figure 3A). As the primers used in this study (mlas/mcrA-rev) can amplify *mcrA* and *mrtA* genes (Steinberg and Regan, 2009), the presence of large number of sequences (32.5%) related to *Methanococcales* (Luton et al., 2002), methanogens carrying *mcrA* and *mrtA* genes, might overestimate the abundance and diversity of the total methanogens. Interestingly, at both similarity cutoffs, *Methanococcales* was represented by a single OTU that is closely related (approximately 95% at the amino-acid level) to *Methanococcus maripaludis*. The occurrences of *Methanomicrobiales, Methanosarcinales* and *Methanococcales* in the *mcrA* sequences were also supported by the results from the analysis of archaeal 16S rRNA gene sequences where the above three methanogenic orders were also the most abundant in the phylum *Euryarchaeota*, which itself represented approximately 35% of the total archaeal sequences (data not shown here).

Among the SRB, the variations among the proportions of OTUs were very low. For instance, the most dominant 70% similarity OTUs (OTU 1, 2, and 3) represented approximately 7, 6 and 6% of the total sequences, respectively (Figure 3B). Phylogenetic analysis indicated that more than 85% of the sequences were related to *Deltaproteobacteria*, suggesting their significant role in the marsh. In the *dsrB* gene sequences, the most frequently detected families of SRB appeared to be *Desulfobacteraceae* (49.2 ± 8.5%) and *Desulfobulbaceae* (29.6 ± 7.8%), whereas sequences related to *Desulfovibrionaceae, Peptococcaceae, Syntrophaceae* and *Syntrophobacteraceae* were detected, but at much lower proportions (total <8%), The dominance of *Desulfobacteraceae* was mainly contributed to by three dominant *dsrB* OTUs that were either closely related to the genus *Desulfosarcina* or uncultured family members (Figure 3B). Interestingly, we detected relatively a large proportion of OTUs (11.3 ± 5.1%) clustered distinctly from the previously isolated *dsrB* phylotypes. This novel cluster formed a distinct deep branch between *Desulfobulbaceae* (with approximately 56.6% amino-acid sequence similarities) and *Syntrophobacteraceae* (with approximately 61% amino-acid sequence similarities) (Figure 3B). An analysis of the total bacterial 16S rRNA gene sequences indicated consistent results with the *dsrB* gene sequence results in that sequences related to the order *Desulfobacterales* were dominant (Figure 7).

### Community distribution patterns with *S. alterniflora* invasion

**Figure 4:**
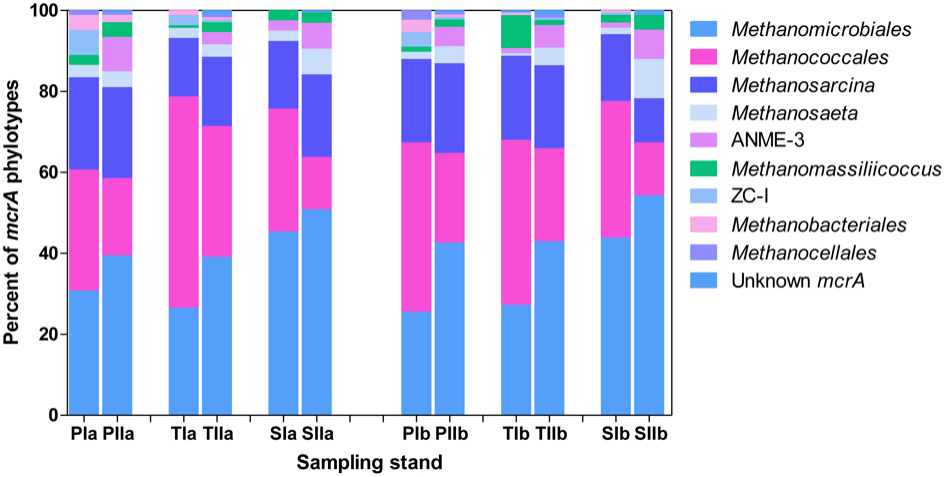
**Proportions of the major *mcrA* phylotypes detected in sediments from *P. australis* (P), *S. alterniflora* (S) and transition (T) stands**. The sample names are as described in Figure 1.

Both invasion- and growth-associated variations in the relative proportions of *mcrA* and *dsrB* phylotypes were analyzed. For methanogens, the orders *Methanomicrobiales, Methanococcales* and *Methanosarcinales* together represented 85–90% of the total *mcrA* sequences (Figure 4). However, their proportion responded differently to the invasion of *S. alterniflora*. For instance, the mean proportions of *Methanomicrobiales*, the most dominant methanogens detected in the salt marsh (representing approximately 33.1 and 44% of the sequences in the spring and summer, respectively), were greater in the *S. alterniflora* stands than in the *P. australis* stands by approximately 58 and 28% in the spring and summer samples, respectively (Figure 4). This might demonstrate the effect of *S. alterniflora* invasion in promoting the proliferation of *Methanomicrobiales*. Almost a reverse phenomenon was observed for the order *Methanococcales* (Figure 4). In the spring, *Methanococcales* represented approximately 38% of the total *mcrA* sequences, but its mean proportions indicated approximately 10% reductions from *P. australis* to *S. alterniflora* stands. In the summer, not only were the *mcrA* sequences related to *Methanococcales* reduced (approximately 20% of the total *mcrA* gene sequences), but higher reductions (approximately 37%) were observed from *P. australis* to *S. alterniflora* stands. Hence, *S. alterniflora* growth appeared to favor *Methanomicrobiales* over *Methanococcales*. However, the two main genera of the order *Methanosarcinales* (*Methanosarcina* and *Methanosaeta*) represented approximately 20 and 24% of the *mcrA* sequences, respectively, although they did not display similar trends with *S. alterniflora* invasion. The mean proportions of *Methanosaeta* increased with *S. alterniflora* invasion. In contrast, the mean proportions of the genus *Methanosarcina* did not display significant variations with either the invasion or growth of *S. alterniflora* (Figure 4). With the exception of ANME-3, most of the rare *mcrA* phylotypes, such as *Methanobacteriales, Methanocellales* and ZC-I, detected in this study dominated the *P. australis* stands. This is consistent with the diversity index results (Table 3) that indicated lower diversity of methanogens in the *S. alterniflora* stands compared with the transition or *P. australis* stands.

**Figure 5:**
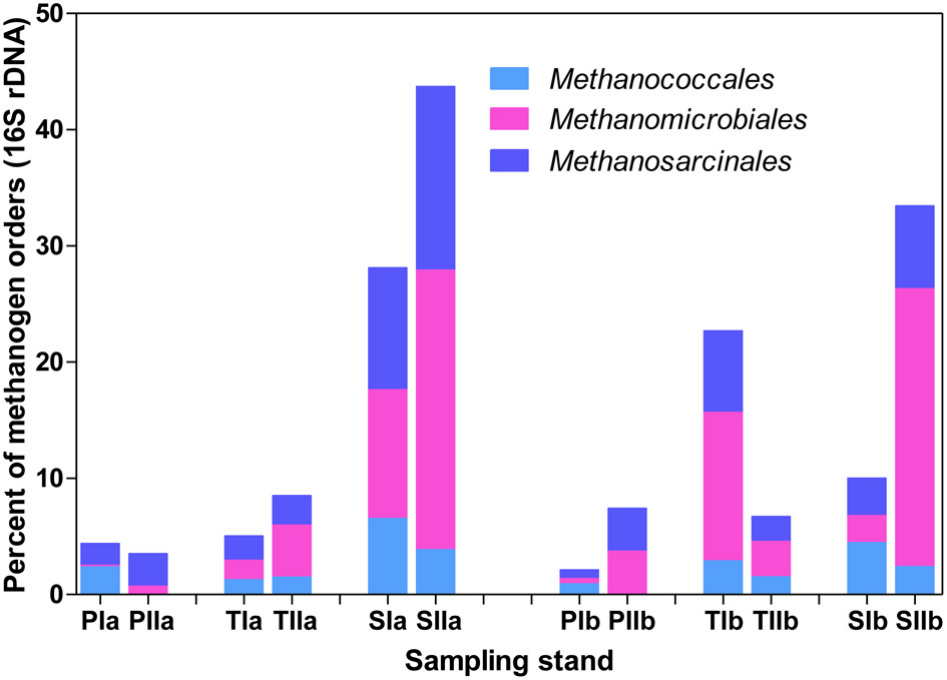
**Proportions of the dominant methanogen orders detected from 16S rRNA gene sequences of *Archaea***. The samples were collected from *P. australis* (P), *S. alterniflora* (S) and transition (T) stands. The sample names are as described in Figure 1.

Trends in the distribution patterns of the dominant methanogen orders revealed that the *mcrA* analysis results were generally consistent with the results of the 16S rRNA gene analysis results, except that *Methanococcales* represented relatively lower proportions of the 16S rRNA gene sequences (Figure 5). The higher proportions of *Methanococcales* in the *mcrA* sequences compared with the 16S rRNA gene sequences might be explained by the likely amplification of both *mcrA* and *mrtA* genes. Hence, methanogens might be slightly overestimated, particularly in the spring samples where *Methanococcales* represented a relatively large proportion of the sequences.

**Figure 6:**
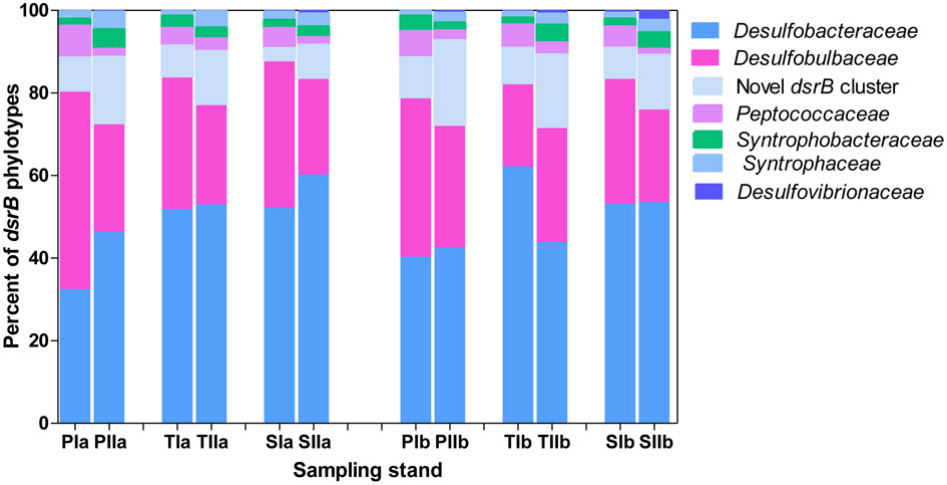
**Proportions of major *dsrB* families detected in sediments from *P. australis* (P), *S. alterniflora* (S) and transition (T) stands**. The sample names are as described as Figure 1.

Based on the frequency of nucleotide sequences recovered from the salt marsh, *dsrB* phylotypes were dominated by *Deltaproteobacteria*, particularly the families of the order *Desulfobacterales* (Figure 6), which represented more than 85% of the sequences. Except for the small increase of its relative proportion at PIa and reduction observed at TIIb, *Desulfobacteraceae* (the most dominant family in the order *Desulfobacterales*) did not display significant change from the spring to the summer. On the other hand, at the S. alterniflora invaded sediments the mean proportion of Desulfobacteraceae increased from approximately 61 to 54% at locations “a” and “b”, respectively (Figure 6). In contrast, the proportion of *Desulfobulbaceae*, the second dominant family in the order *Desulfobacterales*, decreased in the summer (by approximately 45%). However, insignificant change was observed with *S. alterniflora* invasion. Despite the lower proportions (4.0 ± 2.0%), similar trends were observed for the family *Peptococcaceae* (Figure 6) within *Desulfobulbaceae*. Approximately 50% increases were observed from the spring to the summer in the proportions of the novel *dsrB* cluster (Figure 6).

**Figure 7:**
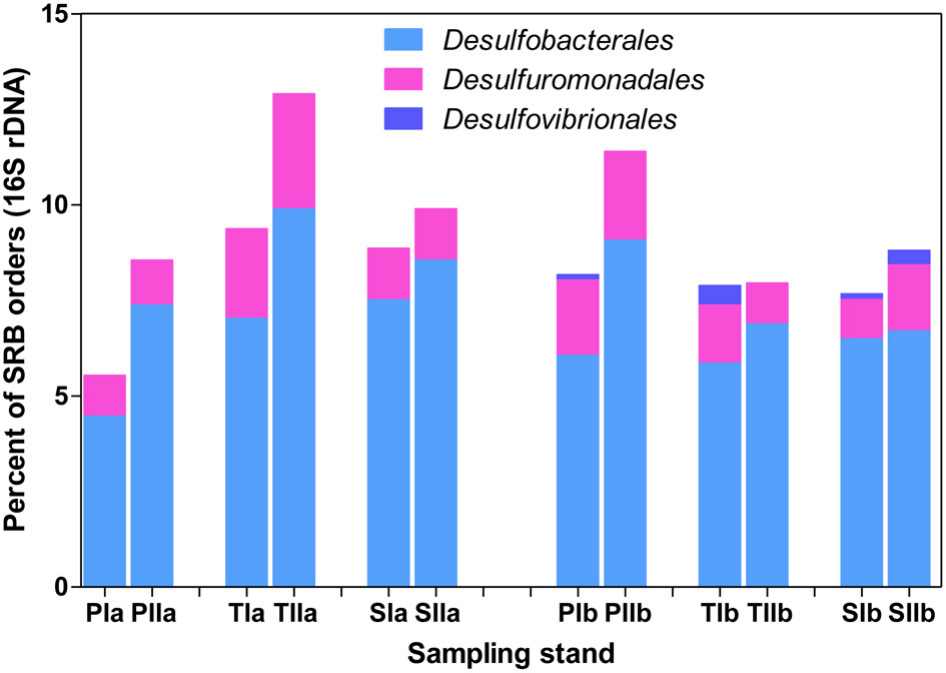
**Proportions of the dominant orders of SRB analyzed from 16S rRNA gene sequences of *Bacteria***. The samples were collected from *P. australis* (P), *S. alterniflora* (S) and transition (T) stands. The sample names are as described in Figure 1.

The abundance patterns of *dsrB* phylotypes were generally consistent with 16S rRNA gene patterns of *Deltaproteobacteria*-related phylotypes (Figure 7). *Desulfobacterales* was represented by 4.5–10% of the total bacterial 16S rRNA gene sequences, whereas *Desulfuromonadales* was represented by 1–5% of the sequences. Similar to *dsrB*, some orders of SRB, such as *Desulfovibrionales*, were represented by very low proportions among the 16S rRNA gene sequences (Figure 7). Generally, insignificant variations in the proportions of SRB phylotypes were observed with the invasion of *S. alterniflora*, although members of *Desulfobacterales* and *Desulfovibrionales* were slightly increased with the growth of *S. alterniflora*.

### Comparison of communities in different stands

**Figure 8:**
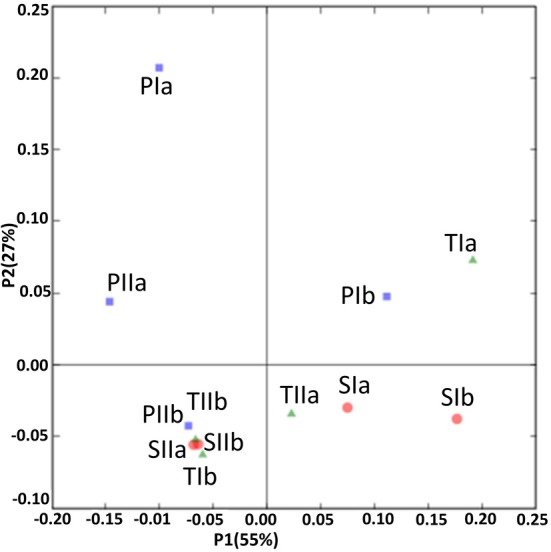
**UniFrac analyses showing the PCA plots of methanogen communities based on sequence abundance data in the *P. australis* (P), *S. alterniflora* (S) and transition (T) stands**. The sample names are as described in Figure 1.

Sediment samples from the different stands were clustered using PCA and environment clustering through weighted normalized UniFrac analysis. With a few exceptions (PIa, TIb and TIIa), PC1 indicated a clear distinction between the methanogen communities of the spring and summer samples (Figure 8). Moreover, most of the communities from the *S. alterniflora*-influenced samples were also clustered distinctly from non-influenced samples in PC2, signifying the variation in the structures of methanogens in the *S. alterniflora* and *P. australis* stands. These variations were clearly supported by the dendrogram clustering of environments where samples from *S. alterniflora* influenced stands, and different growing seasons clustered separately (data not shown).

**Figure 9:**
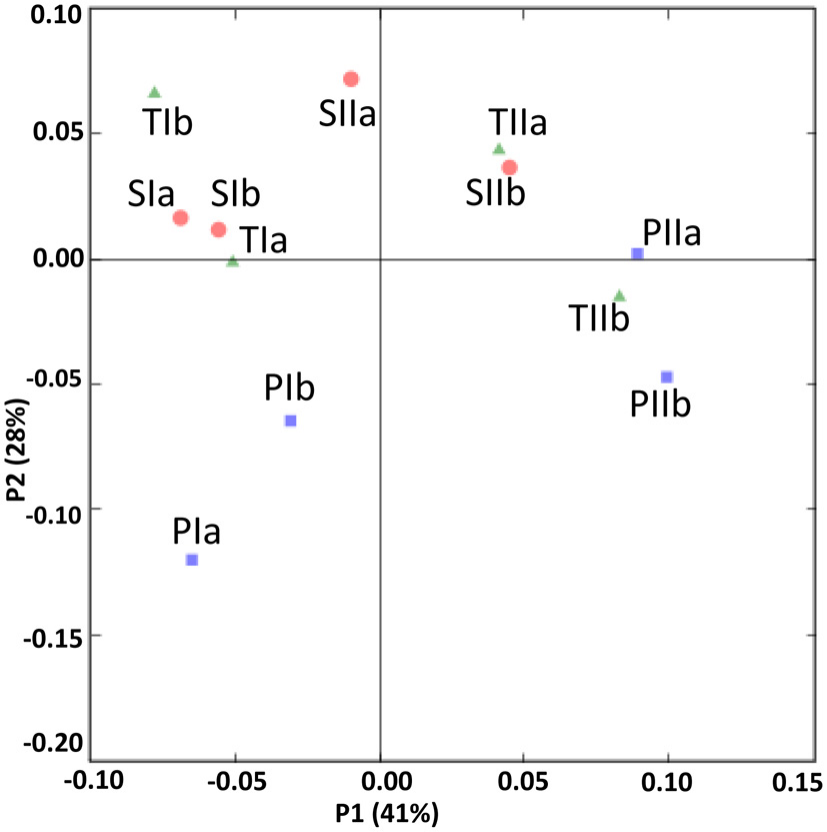
**UniFrac analyses showing the PCA plots of SRB based on sequence abundance data in the *P. australis* (P), *S. alterniflora* (S) and transition (T) stands**. The sample names are as described in Figure 1.

The SRB communities of the spring and summer samples were clustered distinctly along PC1 (Figure 9), which is consistent with the abundance data. The SRB community structures in the samples from the *P. australis* stands and *S. alterniflora* stands distributed separately along PC2 (Figure 9). These results were also supported by the dendrogram of the environment cluster (data not shown).

## Discussion

The extensive expansion of *S. alterniflora* is recognized as one of the major threats to the natural ecology of salt marshes, because the plant can alter the carbon and nitrogen contents of sediments (Turner, 1993; Liao et al., 2007; Page et al., 2010). This, in turn, may influence the carbon and nitrogen cycles in the sediments and the microbes that provide these functions. To understand the impact of *S. alterniflora* invasion on the community structure of methanogens and SRB in the salt marsh sediments of Dongtan, functional gene (*mcrA* and *dsrB*)-based investigations were conducted using samples from stands covered by monocultures of *P. australis, S. alterniflora* and a transition zone where both plants were available.

The higher methane flux rates detected in the *S. alterniflora* stands, particularly during the summer, were not surprising because many studies have already determined the potential of *S. alterniflora* to increase the gas flux rates of environments. This might be associated with the higher density of *S. alterniflora* living biomass, greater gas transportation capacity and substrate stimulation of methane-producing microorganisms (Cheng et al., 2007; Tong et al., 2012). Substrate stimulation of methanogens might be likely, as sediments from *S. alterniflora* stands displayed higher TC and TN levels compared with the native *P. australis* stands. Moreover, increases in the ratio of methane to carbon dioxide in the *S. alterniflora* stands might suggest the invasion of the *S. alterniflora* resulted in greater methane production. This, in turn, could explain the stimulation of methanogens after the invasion. The relatively high soil temperatures detected during the summer could favor the activity of sediment microbes, which might explain the slight reductions of the TC and TN levels of the sediments.

Abundances of methanogens and SRB were increased with the invasion and growth of *S. alterniflora*, which could be related to their available nutrients in the *S. alterniflora* stands. (Schubauer and Hopkinson, 1984; Peng et al., 2011). For instance, Liao and his colleagues (Liao et al., 2008) indicated that the annual litter mass in *S. alterniflora* stands is approximately 22.8% higher than in *P. australis* stands. This abundant *S. alterniflora*-derived organic matter is hydrolyzed and fermented by heterotrophic microorganisms, which can release excess substrates for both methanogens and SRB. The higher abundance of methanogens and SRB in the *S. alterniflora* stands is also consistent with a report that identified higher TC mineralization capabilities of dissolved organic matter derived from *S. alterniflora* compared with *P. australis*-derived matter (Bushaw-Newton et al., 2008). Indeed, the enrichment of sediments with *S. alterniflora* detritus has been shown to fuel the activity of anaerobic microbial communities (Andersen and Hargrave, 1984; Kepkay and Andersen, 1985). Hines and his colleagues (Hines et al., 1999) detected a high number of active sulfate-reducing bacteria (SRB) during the active growth stage of *S. alterniflora* in salt marsh sediments, which may be associated with substrate stimulation of sulfate reducers from root exudates. In contrast, a study on Jiuduansha Island, in the Yangtze River estuary, indicated that the senescence stage of *S. alterniflora* favors the richness and abundance of SRB (Nie et al., 2009), which is likely associated with litter decomposition-related substrate stimulation of SRB. Despite contrasting reports, it is possible to argue that the presence of *S. alterniflora* in environments can alter the natural abundance and activity of SRB. In addition to the relative contribution of *S. alterniflora* to the total carbon and nitrogen levels of sediments (Moran and Hodson, 1990; Liao et al., 2007; Peng et al., 2011), *S. alterniflora* tissues are important sources of trimethylamine (Cavalieri, 1983). Trimethylamine can be a noncompetitive substrate for methanogens. Moreover, acetate might be released from the root exudates or tissue decompositions could be used by SRB and methanogens (King, 1984; Watkins et al., 2012). Temperature is also one of the important factors controlling the growth of methanogens (Zeikus and Winfrey, 1976; Turetsky et al., 2008; Liu et al., 2010), so it is reasonable to conclude that higher abundances of methanogens and higher methane flux rates were detected during the summer and positively correlated with the soil temperature.

Although the abundances of methanogens were higher in the *S. alterniflora* stands of both seasons, their diversities were lower in the *S. alterniflora* stands compared with the *P. australis* stands. Hence, *S. alterniflora* invasion-related abundance increases might not have contributions from all members of the methanogens, and *S. alterniflora* might select methanogen communities. Although the proportions of *Methanomicrobiales* were increased with *S. alterniflora* invasion, *Methanococcales* and many of other rare *mcrA* phylotypes (e.g., *Methanobacteriales, Methanocellales* and ZC-I) were reduced, which might explain the lower diversity of methanogens in the *S. alterniflora* stands. *Methanomicrobiales* and *Methanococcales* (hydrogenotrophic methanogens) unexpectedly dominated the salt marsh (>60%), although they can be easily outcompeted by SRB (Oremland et al., 1982). However, the availability of excess substrates in such productive environments (Schubauer and Hopkinson, 1984; Peng et al., 2011) might reduce the competition and support the growth of both microbial groups. Interestingly, *Methanococcales* was represented by the most dominant OTU (OTU1, approximately 32.5%). Phylogenetic analysis indicated that it is closely related (96%) to *Methanococcus maripaludis*, a methanogen that is commonly distributed in marine and salt marsh sediments (Jones et al., 1983). The reasons for such dominance by a single methanogen species are not clear, but the extremely fast-growing nature of this mesophilic methanogen (Jones et al., 1983) could be triggered by the availability of excess substrates in sediments. On the other hand, the proportion of *Methanosarcina* did not display significant variation with the invasion and growth of *S. alterniflora*, which could be associated with their metabolic flexibility to utilize different substrates (King, 1984; Lyimo et al., 2000). However, the proportion of the genus *Methanosaeta*, strict acetate utilizers, was much lower in the spring but significantly increased with the growth of plants in the summer, indicating the availability of acetate increased with the growth of plants, particularly in the *S. alterniflora* stands where the microbes displayed marked increases.

The current study also revealed that more than 80% of the *dsrB* phylotypes that were detected were members of the order *Desulfobacterales.* These microbes are nutritionally versatile and can oxidize acetate and other organic compounds (Muyzer and Stams, 2008). The SRBA spectrum of substrates from the root exudates and decomposition of *S. alterniflora* and *P. australis* tissues could be available for these diverse SRB (Nie et al., 2009). Except for the small changes in *Desulfobacteraceae*, the proportions of *dsrB* families in both sampling seasons generally displayed minor variations with *S. alterniflora* invasion. The reason for such insignificant variation was not clear; however, nutrients that could be released from *S. alterniflora* tissue decomposition or root exudates might support most SRB phylotypes. The large numbers of novel *dsrB* OTUs that were clustered distinctly between the families *Syntrophobacteraceae* and *Desulfobulbaceae* might offer a clue into the presence of novel SRB types in the tidal salt marsh. Although the physiology of these novel sulfate-reducing phylotypes cannot be speculated about at this time, they could grow with both plant types, particularly in the stands of *P. australis*.

In conclusion, the invasion of *S. alterniflora* in the salt marsh sediments of Dongtan might support the proliferation of methanogens and SRB. However, significant impacts were only observed on the diversity of methanogens.

### Conflict of interest statement

The authors declare that the research was conducted in the absence of any commercial or financial relationships that could be construed as a potential conflict of interest.
